# Hemagglutinating Encephalomyelitis Coronavirus Infection in Pigs, Argentina

**DOI:** 10.3201/eid1403.070825

**Published:** 2008-03

**Authors:** Maria A. Quiroga, Javier Cappuccio, Pablo Piñeyro, Walter Basso, Gastón Moré, Mariana Kienast, Sergio Schonfeld, José L. Cáncer, Sandra Arauz, María E. Pintos, Mariana Nanni, Mariana Machuca, Norio Hirano, Carlos J. Perfumo

**Affiliations:** * Universidad Nacional de la Plata, La Plata, Argentina; † Centro de Investigaciones en Ciencias Veterinarias, Castelar, Argentina; ‡ Private practice, Roque Perez, Argentina; § Iwate University, Morioka, Japan

**Keywords:** porcine hemagglutinating encephalomyelitis coronavirus, epidemiology, pathology, immunohistochemistry, RT-PCR, genomic sequence, dispatch

## Abstract

We describe an outbreak of vomiting, wasting, and encephalomyelitis syndrome in piglets in Argentina, caused by porcine hemagglutinating encephalomyelitis coronavirus (PHE-CoV) infection. Diagnosis was made by epidemiologic factors, pathologic features, immunohistochemistry, reverse transcription–PCR, and genomic sequencing. This study documents PHE-CoV infection in South America.

Porcine hemagglutinating encephalomyelitis (PHE) is an infectious disease that primarily affects pigs <3 weeks of age (*1*). The disease is caused by PHE coronavirus (PHE-CoV) (*2*), which comprises a single strain and is the only known neurotropic CoV for pigs (*3**–**5*)*.*

PHE-CoV was first isolated in Canada from the brains of suckling piglets with encephalomyelitis (*6*); it was later isolated in England from piglets exhibiting vomiting, anorexia, and depression, where it was named “vomiting and wasting disease” (VWD) (*7*). Neurologic and digestive characteristics of the disease were experimentally reproduced in pigs by using the same field virus isolate (*8*). The infection has been reported in the major pig-raising countries of Europe, Asia, and North America, where it seemed to be endemic with no clinical outbreaks (*7*,*9*,*10*).

Presumptive diagnosis can be made by correlating epidemiologic data, age susceptibility information, and disease course with histopathologic findings (*3*,*10*). For definitive diagnosis, the virus should be isolated and identified (*3*). Currently, immunohistochemical (IHC) tests for PHE-CoV or molecular tools such as reverse transcription–PCR (RT-PCR) enable specific CoV RNA sequences to be detected from infected tissues (*11*,*12*). We describe a PHE-CoV outbreak in an intensively managed farm in Argentina as well as the techniques applied for diagnosis.

## The Study

The farm was a 3-site herd with a total of 6,000 sows. At the time of the outbreak, 55% of breeder stock were gilts or first- or second-parity sows. Site 1 comprised 20 gestation barns and 19 farrowing barns, site 2 (nursery) comprised 9 barns, and site 3 comprised growing and fattening barns.

The outbreak began on August 8 and ended on August 23, 2006. Of 19 farrowing barns, 10 (52.6%) were affected. Total proportion of deaths in pigs that had not been weaned was 16.9% (1,226 dead pigs); an estimated 12.6% of pigs that died had suspected PHE-CoV infection (913 animals).

**Figure 1:**
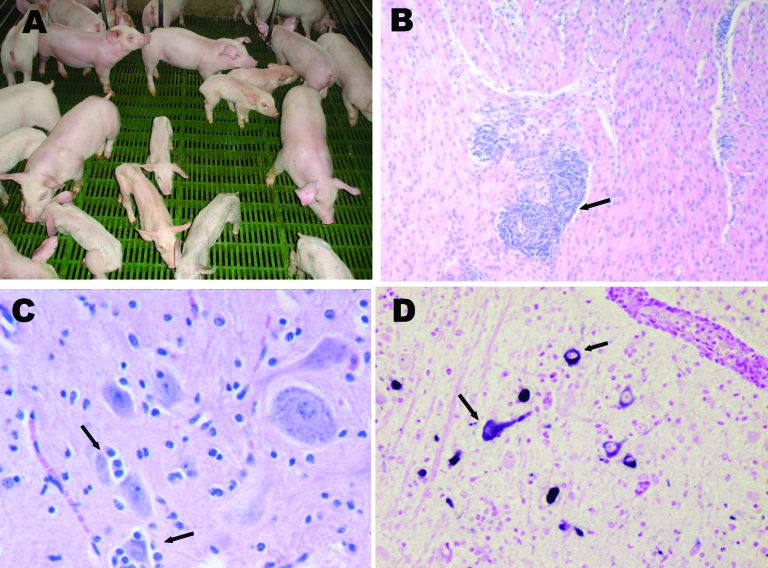
A) Nursery piglets showing clinical signs compatible with porcine hemagglutinating encephalomyelitis coronavirus (PHE-CoV). Nonaffected pigs of the same age are also shown. B) Muscle layer of stomach from affected piglet showing perivascular cuffing (arrow); hematoxylin-eosin stain, magnification ×100. C) Brainstem from affected piglet showing satellitosis (arrows) and gliosis; hematoxylin-eosin stain, magnification x400. D) Brainstem from affected piglet showing positive label of neuron perikarion (arrows); nitroblue-tetrazolium imunohistochemical stain, magnification x400.

Clinical signs were observed in pigs >4 days of age and consisted of vomiting, listlessness, pallor, and dehydration. Neurologic signs such as abnormal gait, dullness, inability to eat, tremors, and nystagmus were observed in some animals. Vomiting and wasting occurred in 27.6% of pigs <1 week of age and gradually declined to 1.6% in pigs 3 weeks of age (mean 13.6%). Twenty-nine percent of weaned pigs housed in nursery barns that received affected animals from site 1 showed wasting (Figure 1, **panel A**), and the proportion of deaths was 15%–40%. In total (sites 1 and 2), 3,683 piglets died or were euthanized.

Postmortem examinations were performed on 16 affected piglets, 2–11 days of age. Samples submitted for histopathologic examination included brainstem, trigeminal ganglia, tonsils, pyloric gland area of the stomach, jejunum, ileum, lymph nodes, heart, spleen, liver, kidneys, and lung.

Selected paraffin sections of brainstem from 5 piglets that had characteristic microscopic lesions were examined for PHE-CoV antigen by IHC tests with an anti PHE- CoV-67N strain mouse antibody; the samples were diluted 1:1,000 and incubated overnight at 4°C. Samples were then labeled with biotinylated conjugated anti**-**mouse immunoglobulin G (IgG) goat antiserum at room temperature for 2 h. The color reaction was detected by alkaline phosphatase and 5-bromo-4-chloro-3-indoyl phosphate by using nitroblue-tetrazolium and true red as cromogens.

RNA was isolated with a commercial kit (RNeasy, QIAGEN GmbH, Hilden, Germany), from brain samples of 7 symptomatic piglets (6–11 days of age) that had nonsuppurative encephalomyelitis, from 1 asymptomatic piglet, and from a PK-15 cell culture suspension inoculated with a pool of tissues from 1 symptomatic piglet. Ribonuclease-A (RNase)–free water was used as negative control. The RT-PCR reaction was performed immediately after RNA isolation by using the specific primer pair for CoV, Cor-FW 5′→3′ (DNA) ACTCAAATGAATTTGAAATATGC, and Cor-RV 5′→3′ (DNA) TCACACTTTGGATAA TCCCA that amplifies a 251-bp fragment of the polymerase gene (*11*). The reaction was performed in a total volume of 50 μL containing 2 μL RNA extract, 10 μL 5× QIAGEN One-Step RT-PCR buffer, 2 μL dNTPs mix (final concentration of 400 μmol/L of each dNTP), 1.8 μL QIAGEN One-Step RT-PCR Enzyme Mix, 4 μmol/L of each primer and RNase-free water to 50 μL. The reaction was conducted in a thermal cycler (PCR Sprint Thermo Electron Corp., Waltham, MA, USA) with an initial reverse transcription at 50°C for 30-s activation at 95°C for 15 s, 40 cycles of amplification (30 s, at 94°C, 30 s at 50°C, and 1 min at 72°C), and a final extension step at 72°C for 10 min.

The amplicons were purified by using the QIAquick PCR purification kit (QIAGEN) and sequenced on a MegaBase 1000 DNA sequencer (GE Healthcare, Chalfont St. Giles, UK). The obtained sequence was analyzed by using NCBI BLAST (www.ncbi.hlm.hig.gov).

Virus isolation was attempted by inoculation of PK-15 and SK-K cells with brains and tonsils from 5 pigs positive for PHE-CoV by RT-PCR. Five blind passages were performed at 7-day intervals. IHC testing was also performed on SK-K cells.

Microscopic changes were observed in samples taken from 5- to 11-day-old affected pigs. The most remarkable changes were perivascular cuffing around Meissner and Auerbach ganglia in the muscle layer of stomach (Figure 1, **panel B**), ganglioneuritis in the trigeminal ganglion, and nonsuppurative encephalomyelitis (Figure 1, **panel C**). PHE-CoV–positive neurons were found in the brainstem (Figure 1, **panel D**) and trigeminal ganglion.

**Figure 2:**
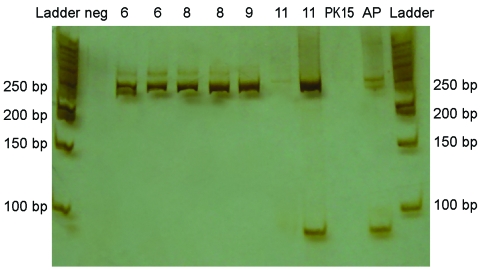
Polyacrylamide gel and silver staining of reverse transcription–PCR products from brains of piglets infected with porcine hemagglutinating encephalomyelitis coronavirus. Amplicons of ≈250 bp were found in brain samples from pigs 6, 8, 9, and 11 days of age. Neg, negative control (water + mastermix); PK15, amplification of PK15 cells inoculated with brain and tonsil from affected piglet; AP, asymptomatic piglet; and Ladder, 50-bp Fermentas.

RT-PCR analysis showed a product of the expected size for CoV (≈250 bp) in all analyzed brain samples. No amplification was observed in inoculated PK 15 cells (Figure 2). The relationship between clinical course, lesions, and IHC and RT-PCR results is shown in the Table. A constant 116-nt sequence was obtained for all products amplified from symptomatic piglets and submitted to GenBank (accession no. EF602436). The sequence showed a 95% identity with the complete genome of PHE-CoV strain VW572 (accession no. DQ 011855) and with PHE-CoV RNA-directed RNA polymerase gene (accession no. AF 124988). Detection of amplicons of ≈250 bp with “pancoronavirus” primers in brain samples is highly suggestive of the presence of PHE-CoV. Sequence analysis confirmed this observation. Cytopathic effects were not observed, and PHE-CoV antigens were not detected in inoculated cells.

**Table:** Clinical signs and results of histopathologic examination, IHC testing, and RT-PCR from piglets affected by PHE-CoV*

Organ	Days old (days with clinical signs)	IHC	RT-PCR	Histopathologic diagnosis
Brain stem	6 (2)	**+**	**+**	Encephalitis
Brain stem	8 (4)	**+**	**+**	Encephalitis
Trigeminal ganglion	8 (4)	**+**	**+**	Focal ganglioneuritis
Brain stem	8 (4)	ND	ND	Encephalitis
Spinal cord	9 (5)	ND	+	Myelitis
Tonsil	9 (5)	ND	ND	Necrotizing tonsilitis
Brain stem	9 (5)	ND	ND	Meningoencephalitis
Medulla oblongata	11(6)	**+**	**+**	Meningoencephalitis

*IHC, immunohistochemical; RT-PCR, reverse transcription–PCR; PHE-CoV, porcine hemagluttinating encephalomyelitis coronavirus; ND, not done; +, positive.

## Conclusions

From an epidemiologic standpoint, the clinical course of the disease (3 weeks), age of affected pigs (<3 weeks), and clinical signs were in agreement with those of VWD caused by PHE-CoV (*3*,*8*,*13*). On PHE-CoV–endemic farms, immune sows apparently provided immunity to their offspring through colostrum (*3*), and clinical disease seldom occurred. In our study, because the gilt pool was so large, a nonimmune subpopulation very likely existed and might have acted as a potential source of virus multiplication. The severity of clinical signs such as vomiting, emaciation, wasting, and death was greater than that previously reported (*10*). Factors that might have enhanced the clinical manifestations of the disease were a nonimmune population and the winter season (*3*,*8*,*10*).

Pensaert (*3*) reported perivascular cuffing and neuronal degeneration at the intramural nervous plexus of the stomach in pigs showing VWD. In addition, Pensaert (*3*) described nonsuppurative encephalomyelitis in 70%–100% of animals showing neurologic signs and in 20%–60% of animals with VWD syndrome. In our study, nonsuppurative encephalomyelitis was found in 50% of infected animals. Our IHC results agreed with findings reported by others in which CoV antigen was detected only in neurons (*8*,*14*,*15*). Detection of amplicons of ≈250 bp in brain samples was highly suggestive of PHE-CoV because this is the only known neurotropic CoV for pigs (*4*). In addition, results observed in samples of nervous tissue processed for IHC, RT-PCR, and sequence analysis added further evidence of the precise causal agent of the current outbreak. This report documents the emergence of PHE-Cov in South America.
